# Graded Exercise Therapy Guided Self-Help Trial for Patients with Chronic Fatigue Syndrome (GETSET): Protocol for a Randomized Controlled Trial and Interview Study

**DOI:** 10.2196/resprot.5395

**Published:** 2016-06-08

**Authors:** Lucy V Clark, Paul McCrone, Damien Ridge, Anna Cheshire, Mario Vergara-Williamson, Francesca Pesola, Peter D White

**Affiliations:** ^1^ Centre for Psychiatry Wolfson Institute of Preventive Medicine Queen Mary University of London London United Kingdom; ^2^ King's Health Economics Health Service and Population Research Department King's College London London United Kingdom; ^3^ Faculty of Science and Technology University of Westminster London United Kingdom; ^4^ Kent & Medway NHS and Social Care Partnership Trust Kent and Medway CFS/ME Service Maidstone United Kingdom; ^5^ Centre for Cancer Prevention Wolfson Institute of Preventive Medicine Queen Mary University of London London United Kingdom

**Keywords:** Fatigue, Chronic Fatigue Syndrome, Myalgic Encephalomyelitis, Secondary Care, Graded Exercise Therapy, Self-Help, Guidance

## Abstract

**Background:**

Chronic fatigue syndrome, also known as myalgic encephalomyelitis (CFS/ME), is characterized by chronic disabling fatigue and other symptoms, which are not explained by an alternative diagnosis. Previous trials have suggested that graded exercise therapy (GET) is an effective and safe treatment. GET itself is therapist-intensive with limited availability.

**Objective:**

While guided self-help based on cognitive behavior therapy appears helpful to patients, Guided graded Exercise Self-help (GES) is yet to be tested.

**Methods:**

This pragmatic randomized controlled trial is set within 2 specialist CFS/ME services in the South of England. Adults attending secondary care clinics with National Institute for Health and Clinical Excellence (NICE)-defined CFS/ME (N=218) will be randomly allocated to specialist medical care (SMC) or SMC plus GES while on a waiting list for therapist-delivered rehabilitation. GES will consist of a structured booklet describing a 6-step graded exercise program, supported by up to 4 face-to-face/telephone/Skype™ consultations with a GES-trained physiotherapist (no more than 90 minutes in total) over 8 weeks. The primary outcomes at 12-weeks after randomization will be physical function (SF-36 physical functioning subscale) and fatigue (Chalder Fatigue Questionnaire). Secondary outcomes will include healthcare costs, adverse outcomes, and self-rated global impression change scores. We will follow up all participants until 1 year after randomization. We will also undertake qualitative interviews of a sample of participants who received GES, looking at perceptions and experiences of those who improved and worsened.

**Results:**

The project was funded in 2011 and enrolment was completed in December 2014, with follow-up completed in March 2016. Data analysis is currently underway and the first results are expected to be submitted soon.

**Conclusions:**

This study will indicate whether adding GES to SMC will benefit patients who often spend many months waiting for rehabilitative therapy with little or no improvement being made during that time. The study will indicate whether this type of guided self-management is cost-effective and safe. If this trial shows GES to be acceptable, safe, and comparatively effective, the GES booklet could be made available on the Internet as a practitioner and therapist resource for clinics to recommend, with the caveat that patients also be supported with guidance from a trained physiotherapist. The pragmatic approach in this trial means that GES findings will be generalizable to usual National Health Service (NHS) practice.

**Trial Registration:**

International Standard Randomized Controlled Trial Number (ISRTCTN): 22975026; http://www.isrctn.com/ISRCTN22975026 (Archived by WebCite at http://www.webcitation.org/6gBK00CUX)

## Introduction

### Background

Chronic fatigue syndrome, also known as myalgic encephalomyelitis (CFS/ME), is a condition characterized by chronic disabling fatigue, which is not better explained by an alternative diagnosis [1-3]. The prevalence of CFS/ME in the population is between 0.4 and 2.5% [3-5]. A working group, reporting to the Chief Medical Officer for England, concluded the following: "CFS/ME is a relatively common clinical condition, which can cause profound, often prolonged, illness and disability, and can have a substantial impact on the individual and the family" [4]. The prognosis is poor with a median of 7% recovering without treatment [6].

The National Institute for Health and Clinical Excellence (NICE) guidelines recommend that patients with CFS/ME are offered GET (or cognitive behavior therapy [CBT]) [7]. In support of this guidance, two systematic reviews showed no harm to patients from GET [8,9]; however, it was concluded that larger trials should be completed to confirm the recommendations. The PACE trial (pacing, graded activity, and cognitive behavior therapy: a randomized evaluation) is the largest ever trial testing GET and CBT for CFS with 641 secondary care patients recruited [10]. Specialist medical care (SMC) alone was compared with SMC plus one of three therapies (adaptive pacing therapy, CBT, or GET). GET was found to be an effective and safe treatment, with 82% being satisfied with GET [11]. In contrast to this research, surveys of ME charity members have suggested that GET is perceived as a harmful and unacceptable treatment. In a recent survey, 56% of those who had received GET reported feeling “worse” afterwards, with 53% reporting GET to be unacceptable [12]. Specialist therapist-delivered GET is also intensive and expensive, with up to 15 sessions required over a 3 to 6-month period [9,11], and United Kingdom (UK) National Health Service (NHS) access is often poor and with long waiting lists [13]. An effective and safe guided self-help GET approach would be helpful to all involved.

Self-help approaches can benefit patients with chronic fatigue in the community [14]. In one study, fatigued patients attending their GP were given an educational CBT self-help booklet [15], complemented by 15 minutes of advice from a research nurse. After 3 months, patients who received the booklet were significantly less fatigued than the usual care group. This booklet has since been used in another primary care trial in which fatigued patients were offered 6 sessions of therapy with a trained GET or CBT therapist at their GP surgery or given the booklet without guidance as an alternative to usual care [16]. A third of patients receiving the booklet significantly improved compared to half receiving the face-to-face therapies.

However, these two studies recruited patients from primary care, and thus included those with less disabling fatigue than that found in secondary care patients. In another study, a guided CBT self-help treatment was tested in secondary care patients with CFS/ME [17]. In this trial, significant decreases in both fatigue and disability were observed in the guided self-help group compared to the waiting list control. A clinically significant improvement in fatigue (27% versus 7% of waiting list patients) and 6-point difference in mean SF-36 physical functioning subscale scores between groups at follow-up resulted. However, problems with respect to engagement and acceptability of psychotherapeutic approaches such as CBT for patients with CFS/ME have been reported [4].

Self-management techniques have consistently been shown to result in substantial improvements in patients with a range of mental illnesses [18-21] and physical illnesses [22,23]. A US study showed that patients with CFS/ME seemed to prefer self-management approaches, particularly social support services [24]. However, no study has investigated guided GET self-help for CFS/ME. In the UK, patients report difficulties accessing specialist services either because there is no local service, because of long waits for referral and/or subsequent treatment, or because they are too ill to travel to the clinic [25]. Approximately 40% reported waiting 6 months to see a specialist. Thus, effective guided self-help could open up a more accessible treatment option for many CFS patients and might make face-to-face therapy either unnecessary, or reduce the need for a full course of therapy.

### Rationale and Piloting

Physiotherapists trained to deliver GET within the PACE trial developed and tested the GES guide. It was developed so that diagnosed patients attending clinics could help themselves using such an approach with advice from their specialist clinician and therapist. The booklet was extensively piloted by secondary care CFS/ME patients and reviewed by several specialists in CFS/ME and a professor of physiotherapy. This led to significant revisions, and review, before “translation” of the guide into lay language by a professional editor.

### Hypotheses

First, we will test the null hypothesis that GES plus specialist medical care (SMC) will be no more effective at improving either physical disability or fatigue than usual SMC alone, as shown by no statistically significant difference between the two arms of the trial 12 weeks after randomization. Second, we will test the hypothesis that GES will be acceptable to patients diagnosed as having CFS/ME in specialist secondary care clinics, as demonstrated by less than 25% of eligible patients declining participation in the trial, and more than 75% of those participating being satisfied with the approach. Third, we will assess the difference in the number of participants suffering serious adverse effects, serious adverse reactions, or a serious deterioration across the study arms.

We will also assess whether there is a statistically significant difference in cost-effectiveness between the two interventions, although this is not a hypothesis.

## Methods

### Study Design

This is a pragmatic [26] randomized controlled trial of outpatients attending two specialist CFS/ME secondary clinics who have been diagnosed by a specialist doctor as having CFS/ME and referred for practitioner-delivered therapy. All participants will be on a waiting list for therapist-delivered treatment. Standard medical care (SMC) will be compared with SMC plus GES.

### Participants

Adults aged 18 and over will be recruited after assessment at two CFS/ME specialist clinics in the United Kingdom (UK): one at St Bartholomew’s Hospital (East London Foundation NHS Trust) and the other in Kent (Kent and Medway NHS and Social Care Partnership Trust). These services each provide assessment and/or treatment for approximately 125 new adults each year, referred mainly from general practitioners. Adults are given a diagnosis after a clinical assessment, with a physical and mental state examination, and screening blood tests according to the guidelines produced by the National Institute for Health and Care Excellence [7]. Approximately 57% of adults referred to specialist CFS services have CFS/ME [27] based on one of three possible criteria [1,2,7]; however, they must meet NICE criteria to be entered into this trial. We chose the NICE criteria to maximize generalizability of the trial to a more representative sample of UK secondary care attendees, as they are more inclusive (requiring a shorter duration and fewer symptoms).

### Inclusion/Exclusion Criteria

Patients will be included if they meet NICE criteria for CFS/ME [7]. To meet these criteria, patients must have clinically evaluated, unexplained, persistent or relapsing chronic fatigue of more than 4 months with a definite onset. Their fatigue needs to have resulted in a substantial reduction in activity, be characterized by postexertional fatigue or malaise and be accompanied by at least 1 of 10 possible symptoms (eg, headaches, muscle and/or joint pain, difficulty sleeping, and concentration problems). Patients meeting these criteria will be referred for treatment in the service as usual and will be asked if they would be willing to be contacted about possible participation in the trial. Patients will not be offered trial participation if they do not speak and read English adequately, have current suicidal thoughts or comorbid psychiatric conditions requiring exclusion, have read the GES guide previously, have had previous GET therapy at one of the trial clinics, or have physical contraindications to exercise.

### Recruitment

Potentially eligible patients identified by the clinician in the initial assessment will be informed about the study and given an information sheet. The clinician will obtain consent from the patient to be contacted by a researcher. If the patient is willing, the researcher will contact the patient and arrange to meet at the clinic or via a Skype™ telephone appointment to provide and discuss further information about the study.

The recruiting researcher will explain the rationale for the study and its design, uncertainties about the effectiveness of the intervention, options available outside of the trial, and the right not to take part in the study or to withdraw at any time up to the analysis. Those willing to take part in the study will be asked to consent to randomization and sign the consent form. If informed consent is subsequently provided, the patient will partake in an assessment. Consent to have guidance sessions recorded will not be required to be included in the trial.

### Randomization

The recruiting researcher will log in to the Web-based automated randomization service/system operated by the UK Clinical Research Collaboration (UKCRC) registered King’s Clinical Trials Unit at the Institute of Psychiatry, King’s College London for the intervention allocation, which will be conveyed to the participant by the recruiting researcher. Allocation will be at the level of the individual, using block randomization with randomly varying block sizes, to preserve allocation concealment. Randomization will be stratified by (1) depression (Hospital Anxiety and Depression Scale; cut-off = 11); (2) severity of disability (SF-36 physical functioning subscale ≤40 and ≥45, which was close to the mean score from all participants in the PACE trial [11]; and (3) by center. This will help to ensure equal proportions of depressed and more severely disabled participants in each treatment arm. Automatic emails will confirm the intervention allocation. If for any reason the randomization service is unobtainable, randomization will be completed during the next working day and the participant will be told of the result by telephone.

### Interventions

#### Standard Medical Care (SMC)

Participants will be informed at the end of their assessment appointment that they have been allocated to SMC and that they should follow the advice of their GP and specialist doctor as usual. They will not have access to the self-help booklet used in the trial. As per usual, specialist doctors will prescribe or advise regarding medication as indicated for symptomatic treatment of associated symptoms (eg, insomnia and pain) and comorbid conditions (eg, depressive illness). These patients will start the therapy to which they have been referred after the endpoint of the trial at 12 weeks or more after randomization, when it becomes available. After completion of trial participation, these patients will also receive a copy of the GES booklet.

#### SMC Plus GES

In addition to receiving SMC, participants in the GES arm will be given a copy of a self-help booklet describing a 6-step program of graded exercise that should take approximately 12 weeks to complete. A physiotherapist will then join the participant for a 30-minute appointment either face-to-face in the clinic or via telephone/Skype™ within 5 working days of randomization. The physiotherapist will explain how the booklet should be used, explain steps 1 to 4 of the booklet, and answer any questions/concerns of participants. The physiotherapist will provide up to 3 further 20-minute telephone or Skype™ support appointments over the next 8 weeks. These 3 follow-up appointments will take place approximately 1 to 2, 4 to 5, and 7 to 8 weeks after the participant’s initial appointment. The physiotherapists will be trained and experienced in delivering GET as a treatment for CFS/ME, and in how to guide and support participants in their use of the booklet without providing additional therapy. The emphasis of guidance/support will be on solutions provided by the booklet and how to apply what is learned, and will follow a support guidance checklist. Contacts with the physiotherapist will be audio-recorded, with informed consent, for training and supervision purposes to ensure that therapists adhere to the guided self-help manual. The GES supervisor will listen to a random sample of the recordings throughout to ensure therapists are maintaining a consistent approach.

*The Guided graded Exercise Self-help (GES) booklet* is based on the approach of GET developed for the PACE trial [11], which was itself based on effective approaches tested in previous trials [9]. GES is also based upon the recommendations made by NICE in 2007 [7]. Patients with CFS/ME attending clinics at St Bartholomew’s and King’s College hospitals in London piloted the booklet. Engaging and encouraging participants to undertake their exercise plans using the GES booklet are cornerstones of the guidance and will be its main focus.

The physiotherapist will use established techniques [28] to maximize engagement and adherence throughout. Participants will be encouraged to approach their graded exercise program using the 6 steps described in the booklet: stabilizing a routine, starting regular stretching, deciding on a goal and choosing a type of physical activity (PA), setting their PA baseline, increasing the duration of PA, and finally increasing the intensity of PA. During each session, the therapist will inquire about progress and answer any questions, with a focus on moving forward to the next step. They will recognize achievements and provide feedback to participants on their efforts, with the aim of increasing motivation and self-efficacy. Near the end of the guidance intervention, the physiotherapist will discuss setbacks. If a participant cannot be contacted by telephone or Skype™, an email will be sent in an attempt to reengage them. After the last guidance session, the physiotherapist will rate the participant’s CGI (health), their adherence to the GES guided support, and their acceptance of the therapy model.

#### Departure from Intended Treatment

To measure departure from intended treatment, participants will be asked at follow-up whether they adhered to the booklet and guidance, and how much PA they undertook in the past week. The number of participants who actively withdraw from either intervention will be recorded. The GES booklet is currently only available from specialist doctors and physiotherapists at specific CFS/ME clinics. For the duration of the trial, the GES booklet will not be available on our websites or patient libraries. Participants who are offered face-to-face therapy before completion of their 12 weeks in the trial, due to an appointment becoming available earlier than expected in the service, will complete their follow-up measures prior to that appointment. This will be considered a “protocol deviation.”

### Assessments and Procedures

#### Criteria for CFS

The research assessment will include evaluation of the operational criteria for the Oxford and Centers for Disease Control and Prevention (CDC) criteria for CFS. Although not eligibility criteria, these will be used in subgroup analyses [1,2]. To determine both excluding and allowable comorbid psychiatric diagnoses, a standardized psychiatric interview (Structured Clinical Interview for DSM-IV Axis I Disorders; SCID) [29] will be conducted by a trained and supervised research assessor.

#### Baseline

The following self-rated inventories will be collected at the first assessment (baseline): 11-item Chalder fatigue questionnaire (CFQ), using Likert scoring [30]; SF-36 physical function short form subscale (SF-36 PF) [31]; Hospital Anxiety and Depression Scale (HADS) [32]; Euroqol Questionnaire (EQ-5D) [33]; Work and Social Adjustment Scale (WSAS) [34]; International Physical Activity Questionnaire (IPAQ) [35]; Tampa Scale of Kinesiophobia-Fatigue (TSK-F) [36]; Client Service Receipt Inventory (CSRI) [37]; and Patient Health Questionnaire (PHQ-15) [38]. Participants will be asked when their CFS/ME started; whether they have ever received GET, CBT, or pacing from a therapist; whether they have ever used any listed self-help resources; and whether they are members of a CFS/ME self-help group. Participants will also be asked about their ethnicity, highest education level, current employment status, whether they have had to reduce/stop work due to their CFS/ME, other health problems, and whether they are taking antidepressant medication for any reason.

#### 12-Weeks Post-randomization

The main outcome end-point will be 12 weeks after randomization, before patients begin their service therapy. We chose 12 weeks as this was about the length of the waiting list for therapy at the St Bartholomew’s CFS service at the time of application for funding. At the end-point, the following information will be collected via questionnaires sent by mail with a stamped addressed envelope: SF-36 PF, CFQ, self-rated Clinical Global Impression of Change (CGI) [39] (this will be done once for overall health and then a second time specifically for CFS), HADS, EQ-5D, WSAS, IPAQ, CSRI, and PHQ-15. Participants will be asked to describe the following: whether they have used any of 4 listed self-help resources since randomization, including the unpublished GES guide [40-42]; how satisfied they are with the help they received; how closely they followed the GES guide; their current employment status and whether they have had to reduce/stop work due to their CFS/ME; any new health problems not already reported; and whether they are taking antidepressant medication for any reason.

#### 12-Months Post-randomization

The primary purpose of the 12-month follow-up will be to obtain the health-economic assessment (see section below); however, we will also collect data on the primary outcome measures and the CGI so that we can assess longer-term physical functioning, fatigue, and change in overall health and CFS. The following information will be collected via questionnaires sent in the mail with a stamped addressed envelope for return: CFQ, SF-36 PF, CGI, EQ-5D, and CSRI.

#### Primary Outcomes

We initially planned to use the SF-36 PF as the primary outcome analyzed as an interval variable collected at 12-weeks postrandomization. The SF-36 PF is scored as the sum of responses to 10 items related to functioning on everyday activities from getting dressed to performing physical activities, each of which is coded 0 for “Yes, limited a lot,” 5 for “Yes, limited a little” and 10 for “No, not limited at all”. This yields a score ranging from 100 for the highest level of perceived physical functioning to 0 for being unable to bathe or dress oneself. The SF-36 PF was to be the sole primary outcome as we were primarily interested in change in physical function.

However, during recruitment we noticed that a significant minority of participants scored close to the mean of the general population (ie, normal physical function) so could be considered recovered even before any intervention [43]. This is because they had substantial reductions in functioning in other domains, such as mental or social activity levels [7].

We therefore added a second primary outcome, the CFQ, which is scored as the sum of responses to 11 items related to physical and mental fatigue, each of which is coded 0 for less than usual,” 1 for “no more than usual,” 2 for “more than usual” and 3 for “much more than usual,” where usual is how they felt the last time they were feeling well. This gives us a symptomatic measure of fatigue. The two primary outcome variables are valid and reliable and have been used in previous CFS trials [9,11]. The ethics committee, Research and Development (R & D), and the trial steering and data monitoring committees approved this change (in June 2013) before any outcome data were formally examined. Because of the change from one to two primary outcomes, we reanalyzed our power calculation and plan to recruit more participants (see section on sample size).

The main secondary outcome measure will be the validated self-rated CGI score, which we will use to measure both change in “CFS” and change in “general health” at the end of treatment, compared with baseline. Each will have 7 possible scores from "very much worse" (score of 7) to "very much better" (score of 1)[39]. Both safety and efficacy can be recorded with this item; we will count scores of 1 and 2 (“very much” and “much” better) as positive outcomes, and scores of 6 and 7 (“very much” and “much” worse) as negative outcomes. Scores of 3-5 (“a little” better, no change, and “a little” worse) will be regarded as no change.

#### Safety Measures and Reporting

For safety outcomes, we will include serious adverse events (SAEs), serious adverse reactions to trial treatments (SARs), and serious deteriorations (SDs). SAEs will be defined according to usual clinical trial definitions (ie, an event that is fatal, life-threatening, or results in or prolongs hospitalization; an increase in severe and persistent disability or incapacity; self-harm; or any other important condition that may require medical or surgical intervention to prevent the above [10] and will be reported to the appropriate authorities in the standard manner. SARs are SAEs that are considered to be a reaction to any trial therapy or drug prescribed. SDs will be defined as any of the following outcomes: CGI scores of 6 and 7, active withdrawal from the intervention due to worsening, or a reduction on the SF-36 PF scale by 10 or more points. Participants will record any nonserious adverse events (NSAEs) in their follow-up questionnaire. An adverse event is defined as any clinical change, disease, or disorder recorded by the participant, whether or not it is considered to be related to the trial or its treatments. Participants will be asked to record whether they believe the adverse event was “related to following the GET guide.” In the event of an adverse event (AE), the center leader will judge the seriousness of the event, and, if judged to be serious, the relationship to a trial supplementary therapy or SMC prescribed treatment, and the expectedness of the event. The trial manager will report all SAEs to the principal investigator (PI) within 24 hours, regardless of the relationship to trial treatment. Reporting of SAEs and SARs will be carried out according to normal regulatory research governance requirements.

After an SAE or SAR, the center leader will make a decision as to whether the participant should be withdrawn from either randomized treatment or from the trial, or if an alteration in their SMC is needed; arrangements will be made for further assessment and management as required. The trial manager will provide the center leader with monthly follow-up reports until resolution. These reports will be communicated to the Data Monitoring and Ethics Committee (DMEC), and other appropriate authorities via the trial manager.

A risk assessment has been undertaken and we have concluded that the therapies are of low risk to participants. NSAEs will be reported en bloc to the DMEC on a regular basis, according to the usual regulatory requirements.

#### Measures Used for Economic Evaluation

Quality of life and function will be measured using the EQ-5D and the WSAS. The EQ-5D will also be used to generate quality-adjusted life years (QALYs) and linked to costs measured using data collected with the CSRI. The IPAQ will determine PA participation before and after the intervention, and the TSK-F will determine beliefs about exercise at baseline. Participants will be asked about their current employment status and whether they have had to reduce/stop work due to their CFS/ME. The physiotherapist will record the number of contacts and the length of each contact, and during the final guidance session, will measure adherence to the GES guided support. Other service use during the trial will be reported by participants completing the CSRI at follow-up, including use of primary and secondary care services, use of other self-help approaches such as the Internet, books or voluntary sector support, medication, therapy outside of the trial, complementary healthcare, and care from family/friends. By accessing clinic notes and relevant electronic databases, we will also collect data on how many therapy sessions participants went on to receive after their trial participation up to one year after randomization.

#### Non-Responders

If a returned form is incomplete, the researcher will contact the patient, usually by telephone, to acquire any missing data. In the event that no outcome data is completed or returned after 2 telephone attempts spaced 1 week apart, the researcher will email or text the participant to ask for answers over the telephone or via email solely for the 2 primary outcomes and the CGI. The researcher will make a file note that the outcome data were collected in this way. If a participant withdraws from the treatment, but not the trial, data will be collected in the usual way. If the participant is not willing to provide all follow-up data, a request will be made to complete only the primary outcome and CGI; if the participant agrees, these will be completed immediately.

### Statistical Considerations

#### Blinding to Outcome Measures

The members of the Trial Steering Committee (TSC), the DMEC, and the trial statistician will be blinded to treatment allocation. The trial manager (TM) and physiotherapists will not be blind to allocation, as they will inform participants of their allocation and deliver the intervention. To further minimize observer bias, outcomes will be self-rated by the participant, and outcome assessments will be coordinated by the TM. The trial statistician will be masked to treatment group until after the main analysis is completed.

#### Sample Size

Our original sample size was based upon the SF-36 PF as our primary outcome; however, the significance level was reduced to 2.5% to accommodate 2 primary outcomes [31]. A large previous trial of CFS/ME using the SF-36 PF (the PACE trial) indicated a baseline mean score of 37 (SD 15) and an outcome score of 48 (SD 21) following 12 weeks of practitioner-led GET, an 11-point increase [11]. Based on these previous findings, and our estimate that GES will be less effective than GET, the sample size calculations are based on the assumption that a mean difference of 8 (SD 18) points between intervention arms at the 12-week follow-up will be a clinically useful difference on the SF-36 PF scale after 12 weeks [11]. Thus, assuming a significance level (alpha) of 2.5% and power of 80%, we require a minimum of 98 participants in each group. This sample size will be upwardly adjusted to allow for loss to follow-up and other compliance issues. Based upon previous trials, we expect about 10% loss to trial follow-up [17]; therefore, we will recruit 109 patients in each group (a total of 218).

Based on a previous trial of GET, in which the difference between baseline and 12 weeks was 5.4 points on the CFQ [11], we assume that a mean difference of 3 (SD 6) points will represent a clinically useful difference at the 12-week follow-up. Hence, assuming a significance level (alpha) of 2.5% and power of 80%, we require a total of 174 patients, which is less than the 218 to be recruited, so adequate power will be achieved.

#### Data Entry and Analysis

Data will be double entered by a dedicated data-entry researcher. The analysis and presentation of the trial will be in accordance with CONSORT guidelines including a flow diagram of enrollment, allocation, follow-up, and analysis [26,44]. We plan to use descriptive statistics to compare characteristics of invited individuals who did or did not agree to take part and eligible individuals who were randomized or not randomized (pending ethical approval). We will also examine differences between trial arms in important baseline participant characteristics.

#### Descriptive Statistics for Primary and Secondary Outcomes

Box plots will be used to assess the data distribution of continuous measures. Descriptive statistics will be broken down by intervention group at baseline and follow-up. Normality of the scales and regression residuals will be explored using diagnostic plots, and if the assumption of normality is violated, the data will be transformed. We will present means and standard deviations for all normally distributed measures, and medians and quartiles for nonnormal measures. Discrete outcomes will be described using both number and percentage.

#### Missing Data

Item-level missing data on the primary and secondary outcome variables at baseline and follow-up will be imputed using mean replacement (prorating). Prorating will be implemented only when less than 20% of item responses per scale are missing. The reasons for missing baseline and follow-up whole-scale data will be summarized using the CONSORT diagram. We will identify baseline characteristics associated with missing data to allow us to impute data to do sensitivity analyses.

#### Analysis for Hypothesis 1

Our primary intention-to-treat analysis will compare the SF-36 PF and CFQ at 12 weeks between groups adjusted using multivariable linear regression analyses [45]. Intention-to-treat (ITT) analyses will be conducted on data from all randomized participants with information at follow-up (ie, modified ITT) regardless of any departure from the allocated treatment arm. We will adjust for our stratification factors (depression, center, and severity of disability) as well as baseline values of outcomes and treatment arm.

A secondary analysis will explore the association between treatment arm and having achieved a clinically useful improvement on the SF-36 PF (ie, an 8-point increase) and the CFQ (ie, a 3-point decrease) using chi-squared analysis.

Analysis of the secondary outcome, the CGI, will compare the proportions scoring “much” or “very much” better (1 and 2), the proportions scoring “a little better,” “no change,” or “a little worse” (3 to 5), and the proportions scoring “much” or “very much” worse (6 and 7) across treatment arms, using an ordinal logistic regression adjusting for our stratification variables.

#### Sensitivity Analysis

Sensitivity analysis will be conducted to assess the impact of missing data on our results. We will estimate whole-scale missing data due to loss to follow-up using multiple imputation by chained equation. This will allow us to conduct a strict ITT for all respondents who took part at baseline. Following recent guidelines, we will also conduct a sensitivity analysis that will take into account the partially nested design of the study and, therefore, assess the potential impact of the “therapist effect” on our results [46]. Further analysis will be conducted to adjust for potential confounders, including sex and age.

A per-protocol analysis will serve as a further sensitivity analysis to investigate the robustness of the conclusions of the primary analysis, following departures from the randomized intervention policies. This will exclude those participants in the control arm who used a GET self-help approach. Subgroup analyses including only those meeting the Oxford or CDC criteria for CFS will also be undertaken. We will analyze moderators of improvement, such as these criteria, but also depression and severity of physical function and fatigue. These subgroup analyses are exploratory and will be interpreted with due caution [47].

#### Analysis for Hypotheses 2 and 3

Chi square will be used to describe any difference in satisfaction (ie, satisfied vs nonsatisfied) across treatment arms and any differences in SAEs, SARs, or serious deterioration (1+ vs none) across treatment arms.

The above plan is what we intend to do; however, the final analyses reported may differ from those planned, allowing for *post hoc* analysis where it is indicated [48]. We will report alternative methods if statistical models do not converge, and omit planned analyses that are superseded, redundant, or no longer of interest. We will report any changes in consequent papers.

#### Economic Evaluation

The economic analysis will take a health and social care perspective. Service use will be combined with appropriate unit costs (eg, from Kent University and NHS Reference Costs) to generate service costs. QALYs gained over the period from baseline to 12-week follow-up will be generated using area-under-the-curve methods from the EQ-5D (using published tariffs from the University of York) combined with costs in the cost-effectiveness analysis. If the intervention group has lower costs and better outcomes than the control group, then the intervention will be seen as “dominant.” If the intervention group has higher costs and better outcomes, we will use incremental cost-effectiveness ratios to identify the extra cost incurred to achieve one extra QALY. Uncertainty around cost-effectiveness estimates will be explored using cost-effectiveness planes (produced from outcome-cost combinations from 1000 bootstrapped resamples). Interpretation will be aided using cost-effectiveness acceptability curves derived using the net-benefit approach with values between £0 and £100,000, placed on a QALY gain so as to include the threshold used by NICE (2007) [7]. We will also conduct similar analyses using costs and the primary outcome measure. However, QALYs are the main measure for the economic evaluation given that thresholds for cost-effectiveness used by NICE exist.

#### 12-Week Follow-Up

The main outcomes, including QALYs, will be measured over the 12-week period from baseline assessment to follow-up. We would expect to see a change in the EQ-5D (which provides us with QALY information) and the WSAS over this time. We will also measure the intervention costs and any short-term impact on the use of other services through the CSRI. We recognize that longer-term impacts, which we are unable to measure may also be important. We will measure serious deterioration using a composite measure including the CGI (“much” or “very much worse”), those who actively withdraw from their intervention, or those whose SF-36 PF score falls by 10 or more points.

#### 12-Month Follow-Up

We will go on to measure how many appointments each participant has with an SMC doctor and with a therapist (and which therapist: psychologist, physiotherapist, occupational therapist, or group therapy) after their trial participation up to 12 months from randomization. We will estimate the costs of the interventions and compare between the groups.

### Data Protection

Participants will be allocated a unique 8-digit identification number made up of a center number, an individual patient number, and patient initials. This number is assigned to the patient and is used on assessment forms prior to transfer of data, so that they are anonymized at source. A list of names and corresponding identification numbers will be kept separately and securely on an encrypted university server. All guided support sessions will be audio-recorded if consent is given, and they will be stored on an encrypted university server.

### Data Monitoring

The DMEC will receive notice of SAEs and SARs for the sample as a whole and per treatment arm. If the incidence of SAEs of a similar type are greater than would be expected in this population, the DMEC will be able to retrieve data according to trial arm, to determine any evidence of excess in either arm. NSAEs will be included in the safety reporting of the completed trial. Suspected unexpected serious adverse reactions (SUSARs) will be reported separately.

### Independent Scrutiny

At the end of the trial, two independent scrutineers will be appointed to consider whether any AE is an SAE and whether any SAE is an SAR. These figures will be reported.

### Compliance

The trial will be conducted in compliance with the Declaration of Helsinki, the trial protocol, Medical Research Council Good Clinical Practice (GCP) guidance, the Data Protection Act (1998), the Multi-centre Research Ethics Committee (MREC), and Local Research Ethics Committees (LREC) approvals and other regulatory requirements, as appropriate. The final trial publication will include all items recommended under CONSORT [49].

### Qualitative Study

#### Purpose

We will undertake a nested qualitative study with a subsample of participants to ascertain patients’ views and experiences of GES, specifically looking for differences in perceptions and experiences between those who improved and worsened with GES.

Both our patient representatives and the TSC suggested using the trial to better understand why patients vary in their responses to graded exercise therapy (GET), particularly by examining engagement and other barriers/facilitators to successful treatment. The best way to gather this knowledge is to undertake a small qualitative study, stratified by both good and poor outcomes. We will conduct one-on-one interviews with participants who have taken part in the active arm of the trial to investigate variations in participant attitudes to, and experiences of, GES. This study will be nested within the time-frame for the main trial.

#### Background Issues

A recent meta-synthesis of qualitative studies uncovered various ways in which people with CFS/ME interpret and experience their illness and their treatments [50]. Themes included wide variability in symptoms, amount of perceived control, and theories about causation and best coping strategies (eg, reducing activities, listening to the body, and balancing activities). Perspectives of patients are also known to change with treatment experience. For instance, if beliefs about the need to avoid exercise and less helpful thought patterns can be addressed (eg, catastrophic thinking), then improvements in fatigue may follow [51]. At the same time, some patient organizations believe that one particular treatment, graded exercise therapy (GET), is harmful to CFS/ME patients [12]. Qualitative research can provide a deeper understanding of the way that participants approach, experience, and give meaning to interventions like GES [52]. There is no published, qualitative research that investigates variation in participant attitudes to, and experiences of, GET in a randomized controlled trial. Yet, the evidence is that patients who benefit from such interventions are likely to approach the intervention differently than those who do not [53]. Our preliminary (unpublished) analysis of free-text feedback from GET participants in the PACE trial [11] suggested different categories of participants. For instance, people differed in their appraisals of GET from positive to negative, and whether they attended more to the learning, support, treatment, and/or contextual issues outside of the trial. However, we do not yet have sufficient data to examine the significance of different styles of patient engagement with GET.

#### Research Questions for the Qualitative Study

The following two research questions will be investigated in the qualitative study: (1) Are there any differences in treatment perceptions and experiences between those trial participants who improved and those who worsened during use of guided self-help based on GET? and (2) What are the implications for the way patients understand “getting better?”

#### Recruitment and Sampling Strategy

We aim to recruit up to 20 participants, all of whom will have received GES. Participants will be stratified into 2 similar-sized groups of either improvement or deterioration (CGI score at 12-week follow-up assessment of “much” or “very much” better versus “much,” or “very much” worse) for comparison [39]. We will seek written informed consent from these 20 participants. Participants will have completed the 12-week follow-up assessment, and will be recruited at least one month later to allow time for them to reflect on their GES and trial experience.

#### Participant Involvement

Participants will undertake a one-off semistructured interview, either face-to-face, by telephone, or by Skype™, depending on their preference.

#### Data Collection

Qualitative semistructured interviews, digital audio-recorded, with fully informed consent, will be conducted with participants. An experienced qualitative researcher will conduct the interviews, using a semistructured approach [54]. By ensuring the same topics are covered in each interview, we will collect comparable data about participants’ experiences of the trial and treatment within each group. In addition, the semistructured approach will allow greater flexibility for participants to highlight their specific concerns, meanings, and priorities, even if not anticipated by the researchers. Topics will include before and after trial well-being, expectations of treatment, understanding of “baseline” and “recovery,” the meaning of exercise, barriers and facilitators to treatment, and any outside influences on trial participation. Thus, we will use an approach suited to collecting a wide range of experiences in each group. We will use face-to-face interviews to collect people’s experiences (eg, in people’s homes, or a university interview room). We will also use less conventional ways to include the perspectives of people who may have trouble participating; for example, recording of interviews over the phone, or the use of Skype™ video calls for interviews. Sample questions are provided in Multimedia Appendix 1.

#### Data Management and Analysis

The audio-recordings from the interviews will be professionally transcribed (a confidentiality agreement will be in place). The transcription will be reviewed against the audio by the researcher for errors and to remove any identifying information, and then returned to the participant to check accuracy and add any clarifying points at the end of the interview, with a deadline of one month to reply.

Thematic analysis (ie, “identifying, analyzing, and reporting patterns within data”) is a basic building block of all kinds of qualitative analysis [55], and will be the approach used for analysis in this study. Data will be inputted and coded in the qualitative data analysis software environment, NVivo. NVivo will aid the coding of themes, organization, and searching of all interviews in both patient outcome categories (improvement vs worsening). The software will enable the comparison of themes across the full range of data (constant comparison, ie, comparing all bits of similar data with each other) [56] so more robust conclusions can be drawn.

While the researcher will drive the analysis, all investigators will be involved in group analytical sessions, debating and clarifying themes, and drafting of reports to arrive at the final analysis.

### Patient and Public Involvement

Patients were significantly involved in the design and piloting of the GES booklet before the trial started. Two patient representatives have also advised us in design of this trial and in the application and amendments to this trial. A patient representative is a member of the TMC, which will meet twice yearly. This individual will therefore be intimately involved in all aspects of this trial, so that we can ensure that this research reflects the needs and views of the patient group. A representative of the Association for Young People with ME will sit on the TSC.

### Ethics and Dissemination

The study was approved on November 23, 2011 (reference 11/LO/1572) by NRES Committee London, London Bridge. Four favorable opinions were provided on May 8, 2012, May 31, 2012, June 27, 2012, and June 20, 2013, for amendments to the study protocol and documents. A fifth amendment, requesting the addition of a nested qualitative study was rejected on 2014 September 1, with the suggestion it should be submitted as a separate study. The qualitative study received a favorable ethical opinion on January 9, 2015 (reference 15/WM/0007) by NRES Committee West Midlands, The Black Country. All patients involved in the study provided informed consent to take part in the study.

The results of the GETSET study will be disseminated to the scientific community, media, relevant charities, and general public. Results will be published in peer-reviewed international journals and will be presented at national and international conferences and symposiums.

## Results

The trial has finished recruiting (first randomization: May 15, 2013; last randomization: December 24, 2014). Follow-up for the main analysis was completed in April 2014 and long-term follow-up in March 2016. Data analysis are underway and the first results are expected to be submitted for publication later this year.

## Discussion

CFS/ME is a relatively common and frequently disabling condition with limited treatment options of proved efficacy available within the NHS. GET is recommended, but access to it is limited, with patients often spending many months waiting for rehabilitative therapy with no help given during that time. The longer someone has CFS/ME, the worse the prognosis [6].

Guided exercise self-help (GES) has never been tested in a randomized controlled trial for CFS/ME. It is important for people with CFS and the NHS to know whether GES is more effective, and cost-effective, when used with SMC, than when SMC is used alone.

If this trial shows GES to be acceptable and safe, and shows a clinically useful difference between arms, then the GES booklet will be made available on the Internet and through other media as a practitioner resource, with the knowledge that it is an acceptable, safe, and effective treatment when supported by advice from a trained therapist. If found to be cost-effective, then it could be recommended for commissioners of services. It could be used as a first line of treatment while patients are waiting for face-to-face specialist practitioner-delivered therapies, such as GET and CBT. Guided self-help exercise could become the first step in a stepped-care approach for CFS/ME in the NHS, by training current physiotherapists to deliver it.

The next trial would be to test this intervention in primary care, which could immediately follow this trial. The secondary care trial is necessary first, to ensure this intervention is effective in those with properly diagnosed CFS/ME, before testing it in those at the primary care level, where the diagnosis is not so well-established [57].

If shown to be an effective treatment, this policy of providing a guided self-help approach to initially stabilize and then increase PA in patients with CFS/ME could also be tested for patients with many other chronic disabling conditions known to respond to practitioner-delivered graded exercise approaches, including arthritis, chronic obstructive airways disease, and diabetes mellitus [58].

## Appendix

Multimedia Appendix 1 Sample questions.

## Abbreviations

CBT: cognitive behavior therapy
CFS: chronic fatigue syndrome
CGI: Clinical Global Impression change score
CFQ: Chalder Fatigue Questionnaire
CSRI: Client Service Receipt Inventory
DMEC: data monitoring and ethics committee
EQ-5D: Euroqol Questionnaire
GES: guided graded exercise self-help
GET: graded exercise therapy
HADS: Hospital Anxiety and Depression Scale
IPAQ: International Physical Activity Questionnaire
ITT: intention to treat
ME: myalgic encephalopathy/encephalomyelitis
NICE: National Institute for Health and Clinical Excellence
NHS: National Health Service
NRES: National Research Ethics Service
PA: physical activity
PHQ-15: Patient Health Questionnaire (15 questions)
PI: principal investigator
QALY: quality-adjusted life years
SAE: serious adverse event
SAR: serious adverse reaction
SCID: Structured Clinical Interview for DSM-IV Axis I Disorders
SF-36 PF: Short Form 36-Questionnaire physical functioning subscale
SMC: specialist medical care
TM: trial manager
TMG: Trial Management Group
TSC: trial steering committee
TSK-F: Tampa Scale of Kinesiophobia-Fatigue
WSAS: Work and Social Adjustment Scale
