# Supplementary material for: Graded Exercise Therapy Guided Self-Help Trial for Patients with Chronic Fatigue Syndrome (GETSET): Protocol for a Randomized Controlled Trial and Interview Study
Source: JMIR Res Protoc. 2016 Jun 8;5(2):e70. doi: 10.2196/resprot.5395 (PMC4917732; doi:10.2196/resprot.5395)
Supplement: Multimedia Appendix 1 [file resprot_v5i2e70_app1.pdf]

Multimedia Appendix 1. Sample Questions

These questions are indicative of the topics discussed, although the researcher will vary the way questions are asked (consistent with semistructured interviewing to improve data collection).

1. If you can think back to immediately before you started your GETSET treatment, can you tell me how the CFS/ME was affecting you at the time? [Prompt: Can you recall the circumstances when you found out about GETSET if you are having difficulty remembering?]
2. Why did you decide to take part in GETSET?
3. What were you expecting from treatment in the trial? [Prompt: How was your experience of the trial different from your expectations?]
4. Did you have any concerns about doing exercise during GETSET? [Prompt: If yes, can you tell me about these?]
5. How was GES explained to you, if at all? What is your understanding of how GET works now? [Prompt: What is your understanding of the theory behind it?]
6. What do you understand is meant by “setting a baseline”? [Prompt: Did you have trouble reaching baseline?]
7. What do you understand is meant by the term “exercise”? [Prompt: How does exercise affect your health?]
8. To what extent did you stick to your GETSET treatment program?
9. How well did GES work for you?
    1. [Prompts: Compared with the way things were before the trial? How well have you felt since the trial finished?
    2. Were there parts of the approach that worked better or worse than others Please explain?]

10. Why do you think GES [did not work/worked] for you?
11. Were there any barrier(s) to doing GET? [Prompt: If yes, can you tell me about these?]
12. Was there anything in particular that helped you to do the GET? [Prompt: Did anything help/ make it easier for you to do GES?]
13. How has your attitude to exercise changed after having been in the trial (if at all)?
14. To what extent do you feel you can get better [recover] from this illness? [Prompt: What does recovery mean to you?]
15. Was there anything important going on in your life at the time of GETSET [Prompt: Could anything else have affected your well-being/participation in GETSET?]
16. Did you see the graded exercise therapy treatment as physical exercise, physical activity, or something else? [Prompt: Understanding of labels].
17. Is there anything else you want to say, that you have not had a chance to say? [Prompt: Learnt anything? Social and practical support?]
